# Alginate encapsulation improves probiotics survival in carbonated sodas and beers

**DOI:** 10.1371/journal.pone.0283745

**Published:** 2023-03-31

**Authors:** Li Ling Tan, Kai Lin Ang, Say Chye Joachim Loo

**Affiliations:** 1 School of Materials Science and Engineering, Nanyang Technological University, Singapore, Singapore; 2 Singapore Centre for Environmental Life Sciences Engineering (SCELSE), Nanyang Technological University, Singapore, Singapore; 3 Lee Kong Chian School of Medicine, Nanyang Technological University, Singapore, Singapore; Sathyabama Institute of Science and Technology, INDIA

## Abstract

Probiotic functionalization of non-dairy beverages has been garnering interest to provide dairy-sensitive populations with greater probiotic product varieties. The addition of probiotics into popularly consumed beverages–carbonated sodas and beers, presents an interesting challenge as the presence of acidic pH, hops-derived compounds, and ethanol have highly deleterious effects. Herein, alginate encapsulation was proposed to improve probiotics viability within sodas and beers. Three probiotics, namely *Lacticaseibacillus rhamnosus* GG, *Escherichia coli* Nissle 1917, and *Bifidobacterium longum* were encapsulated in alginate spheres and exposed to Coca-Cola, 7-Up, Tiger Beer, and Guinness under refrigerated, room temperature and simulated gastric fluid conditions. Results demonstrate that alginate encapsulation significantly improved the viabilities of all three probiotics in various beverages and conditions. Refrigerated storage better preserved probiotic viabilities and reduced the formation of the probiotic metabolic by-product, L-lactate, than at room temperature storage. Findings here could provide beverage manufacturers with a novel way to develop probiotic-sodas and probiotic-beers through encapsulation.

## 1. Introduction

The human gastrointestinal tract (GIT) is inhabited by trillions of microbes which play crucial roles in regulating numerous aspects of human health. In recent years, the importance of gut microbiota has become broadly accepted, and the use of microbiome-modulating ingredients, particularly probiotics, has become increasingly mainstream [1]. Probiotics are “live microorganisms which when administered in sufficient amounts, confer a health benefit on the host” [2]. Presently, probiotics are primarily consumed via probiotic supplements (e.g., pills, tablets, sachets) and probiotic dairy beverages (e.g., yogurt, yogurt drinks, cheese). A growing research interest exists in developing new food and beverage formats with the incorporation of probiotics, to provide consumers with greater probiotic product varieties, as well as cater to dairy-sensitive consumers (e.g., lactose-intolerant, or vegan consumers).

An important criterion for probiotics is that they should remain viable throughout the manufacturing process, storage, and passage through the upper GIT [3]. The incorporation of probiotics in certain food or beverage matrices still presents a challenge currently, as certain food components and their storage conditions can greatly reduce probiotics viability. Carbonated sodas/soft drinks and beers are two such beverage matrices that pose highly deleterious conditions for the survival of probiotics. Carbonated sodas and beers are popularly consumed beverages with global market values of US$ 332 B and US$ 552 B in 2021, with expected compound annual growth rates (CAGR) of 4.50% and 6.81% respectively [4, 5]. Carbonated sodas are known to be highly acidic, with pH values ranging from 2 to 3.5, depending on the type of soda [6]. This acidic pH is due to the presence of acids, such as phosphoric acid and citric acid, which are added to impart distinctive tart and tangy flavors to sodas [6]. Dissolved carbonic acids also contribute to acidity in carbonated sodas. These acids provide bactericidal and fungicidal effects, which under usual circumstances, are preferred by soda manufacturers for their preservative function. However, this low pH property becomes a challenge in attempts to incorporate live probiotic cultures into carbonated sodas. For beers, the main components which affect probiotics viability are ethanol and hops iso-α acids. Ethanol concentration in beer varies between 3–9%, depending on the type of beer. Ethanol is known to have antiseptic properties and inhibits bacterial growth by disrupting membrane stability and denaturing proteins [7]. Hops iso-α acids, which are extracted from hops during wort boiling, are potent antimicrobial compounds that can inhibit Gram-positive bacteria [8]. The antibacterial mechanism of hops iso-α acids has been associated with their role as ionophores, which dissipates the pH gradient across bacterial membranes and reduces proton motive force [9]. Hops iso-α acids also contribute mild acidity to beers. Probiotic bacteria, particularly those which were not isolated from alcoholic beverage or food sources, typically do not have intrinsic resistances to ethanol and hops. As such, while carbonated sodas and beers are popular beverages frequently consumed by the masses, there exists a significant challenge to functionalize these beverages with probiotics.

Alginate encapsulation is a technique that has been widely proposed to reduce the viability losses of probiotics [10–12]. Alginate is a naturally occurring polysaccharide comprising of guluronic acid (G) and mannuronic acid (M) residues, and thus it exists as a polyanion in a solution [13]. It is preferred as a probiotics encapsulation material due to its generally regarded as safe (GRAS) status, edibility, ease of gelation, and low cost [11]. Alginate encapsulation of probiotics is most frequently achieved by an external gelation process, which involves dropping alginate-probiotic suspensions into a crosslinking solution, such as calcium chloride. Crosslinked alginates tend to have spherical bead-like structures similar to “boba” that is popularly consumed in bubble tea. Alginates are known to confer gastric acid-protective effects on entrapped probiotics [12]. In an acidic environment, alginate converts to alginic acid, which sequesters protons from the acidic milieu and achieves a pH buffering effect [14]. Encapsulated probiotics are typically released in the intestine (pH 7), as the higher concentration of phosphate ions in intestinal fluids sequesters cation crosslinkers and causes disintegration of the crosslinked alginate matrix [14]. These useful properties of alginate encapsulation have provided inspiration for this article, and we sought to evaluate if alginate encapsulation by external gelation can improve probiotics viability in carbonated sodas and beers.

There have been few literatures regarding the incorporation of encapsulated probiotics in carbonated sodas and beers. Prior literature mostly discussed the incorporation of alginate-encapsulated probiotics in fruit juices (e.g., orange or apple) [15, 16], the use of probiotics as starter-cultures for beer fermentation [8], or addition of unencapsulated probiotics to low- and non-alcoholic beers [17]. To the best of our knowledge, the addition of alginate-encapsulated probiotics into beers was only once explored by [18], which tested the survival of *Lacticaseibacillus rhamnosus* GG (LGG) in a commercial Lager Beer. In this article, we aim to examine the viability of unencapsulated and alginate-encapsulated probiotics in commercialized sodas and beers (both lager and stout), including Coca-Cola (Coke), 7-Up, Tiger Beer, and Guinness, and evaluate if alginate encapsulation by external gelation can improve probiotics viability in these beverages. Three probiotic strains of different genera, namely *Lacticaseibacillus rhamnosus* GG (LGG), *Escherichia coli* Nissle 1917 (ECN), *Bifidobacterium longum* (BL), were individually tested in the respective sodas and beers under 25°C room temperature, 4°C refrigerated, and 37°C simulated gastric fluid (SGF) exposure conditions. The findings from this study can provide beverage manufacturers with a potential method of incorporating viable probiotics into their products.

## 2. Materials and methods

### 2.1 Materials

LGG was isolated from a purchased Culturelle® LGG probiotic pill (i-Health, Inc., USA). ECN was isolated from a purchased Mutaflor® pill (Pharma-Zentrale GmbH, Germany). BL ATCC 15707 was purchased from ATCC, USA. De Man, Rogosa and Sharpe (MRS) and Tryptic soy (TS) media were purchased from Thermo Fisher Scientific, USA. Luria-Bertani (LB) media and Bacto agar were purchased from BD, USA. Carbonated sodas and beers, including Coca-Cola Classic (The Coca-Cola Company, USA), 7-Up (Keurig Dr Pepper, USA), Tiger Asian Lager (Heineken Asia Pacific, Singapore), and Guinness Foreign extra stout (Diageo, UK) were purchased from local supermarkets (see **S1 Table in S2 File** for their ingredient lists). Protanal CR8133 sodium alginate was purchased from FMC BioPolymer, USA. Glutaraldehyde and sodium cacodylate buffer were purchased from Electron Microscopy Services, USA. All other chemicals used in this experiment were purchased from Sigma Aldrich, USA. Sterilization by autoclaving at 121°C for 15 min was done for every media, agars, chemical, or apparatus prior to use, where necessary.

### 2.2 Methods

#### 2.2.1 Growth and preparation of probiotics

Single colonies of LGG, ECN, and BL were inoculated in sterile MRS, LB, and TS broths respectively and incubated at 37°C for 24 h (aerobic incubation for LGG and ECN, anaerobic for BL) to reach the stationary phase of growth. The probiotics were then adjusted to following designated OD600 values; for LGG, 6 (~ 5×10^8^ CFU/ml), for ECN, 1 (~ 5×10^8^ CFU/ml), and for BL, 1 (~ 7×10^7^ CFU/ml). The probiotics were then washed thrice with 0.9% (w/v) NaCl, with centrifugation at 10,000 xg for 5 min between each wash. Next, the probiotic cell pellets were resuspended in 0.9% NaCl at one-tenth of the original volume to obtain a 10x concentrated probiotic sample. Drop plating on respective agars was performed to determine the initial CFU concentration of various probiotics.

#### 2.2.2 Exposure of unencapsulated probiotics to beverages and SGF

50 μl of washed 10x concentrated LGG, ECN, and BL probiotics were added to 4.95 ml of various beverages (Coke, 7-Up, Tiger Beer, and Guinness) in triplicates, and thoroughly mixed by vortexing. These beverages were incubated at 25°C room temperature or 4°C refrigerated conditions with 50 rpm shaking on an orbital shaker, for up to 14 days, with aliquots retrieved at 1 h, 2 h, 1 day, 2 days, 3 days, 7 days, and 14 days exposure duration. Viable probiotic counts at the respective exposure durations were enumerated by serial dilution and drop-plating.

Various unencapsulated probiotics were also exposed to SGF and beverage combinations, to determine the survival of probiotics in a gastric environment following oral consumption of sodas or beers. SGF was prepared by dissolving 2000 U/ml porcine pepsin in 0.2 M NaCl pH 2 solution, and sterilized by filtration through a 0.45 μm membrane. An equal volume of SGF was added to various beverages to form a beverage-SGF solution. 50 μl of 10x concentrated probiotics were then added to 4.95 ml of various beverage-SGF combinations, and incubated at 37°C, 200 rpm shaking conditions for 2 h. Aliquots were retrieved at 1 h and 2 h exposure time points, and surviving probiotics were enumerated by serial dilution and drop-plating.

#### 2.2.3 Encapsulation of probiotics by extrusion

Probiotics were encapsulated following a similar protocol as [11]. 2.22% (w/v) sodium alginate with 11.11% (w/v) sucrose was dissolved in deionized (DI) water and autoclaved (121°C, 15 min) in advance. The 10x concentrated probiotics suspension was then added in a 1:9 (v:v) ratio with the alginate-sucrose solution and thoroughly mixed, thereby yielding a final 2% (w/v) alginate with 10% (w/v) sucrose with 10^7^–10^9^ CFU/ml of probiotics. The cell suspension was extruded via a syringe pump (KD Scientific, USA) through a 26G needle into 40 ml of sterile 0.1 M calcium lactate crosslinking bath, with a magnetic stirrer kept stirring at 250 rpm. The needle-to-crosslinking bath distance used was 1 cm and flow rate used was 1 ml/min. Alginate-probiotic beads were left to crosslink for 30 min, thereafter, beads were harvested and washed thrice in sterile water to remove any unencapsulated bacteria.

#### 2.2.4 Imaging

The morphology of extruded beads was characterized using light microscopy (Stereo Discovery V8, ZEISS, Germany) and scanning electron microscopy (SEM). Chemical fixation was done for extruded beads prior to SEM imaging. Probiotics-loaded alginate beads were immersed in 2% (v/v) glutaraldehyde in 0.1 M sodium cacodylate buffer pH 7.4 for 1 h. Beads were then rinsed thrice in 0.1 M sodium cacodylate buffer, then thrice more in DI water. Dehydration was carried out by soaking beads in 30%, 50%, 70%, 90%, and 100% (v/v) ethanol sequentially for 5 min each. Ethanol was then removed, and beads were frozen at -80°C, followed by freeze-drying (0.027 bar, -84°C). Freeze-dried beads were observed using a JSM-6360 SEM (JEOL Ltd., Japan) operating in the secondary electron mode. Freeze-dried powders were mounted to a stub with carbon tape and coated with 10 nm of conductive gold prior to imaging.

#### 2.2.5 Exposure of encapsulated probiotics to beverages and SGF

Encapsulated probiotics were exposed to various beverages and beverage-SGF solutions in a similar method as for the unencapsulated probiotics (**Section 2.2.2**). 500 ± 10 mg of alginate probiotic beads were added to 5 ml of beverage/beverage-SGF solutions, and incubated under 25°C, 4°C, or 37°C conditions. Probiotic beads were harvested at 1 h, 2 h, 1 day, 2 days, 3 days, 7 days, and 14 days time points for beverage exposure, and at 1 h and 2 h time points for beverage-SGF exposure. Encapsulated probiotic counts at various time points were enumerated by dissolving beads in 0.05 M sodium citrate followed by serial dilution and drop-plating.

#### 2.2.6 Lactate quantification

Lactic acid is a known by-product of lactobacilli glucose metabolism which can potentially alter beverage taste if accumulated in high concentrations [19, 20]. Lactate concentration was evaluated in various beverages with added LGG. Unencapsulated and encapsulated LGG were added to various beverages in a similar method as described in **Section 2.2.5**, and incubated at 25°C room temperature or 4°C refrigerated conditions, with 50 rpm shaking. Aliquots of beverages were removed at 0 h, 2 h, 24 h, 48 h, 72 h, 7 days, and 14 days exposure duration. The aliquots were filtered via a 0.2 μm membrane, and analyzed using Shimadzu Prominence high performance liquid chromatography (HPLC) (Shimadzu, Japan) equipped with an Aminex HPX-87H organic acid analysis column (Bio-Rad Laboratories, USA). The column was maintained at a temperature of 40°C, and 30 μl of each sample was eluted with filtered, degassed 0.005 M H_2_SO_4_ solvent at 0.6 ml/min. Sodium L-lactate in concentrations of 0.01–50 mg/ml were used as standards (**S1 Fig in S2 File**). The lactate peak was identified at retention time of ~ 12.5 min. Concentrations of lactate produced by LGG in various beverages were calculated by normalizing concentrations with the 0 h baseline.

## 3. Results and discussion

### 3.1 Survival of unencapsulated probiotics in beverage

**Fig 1:**
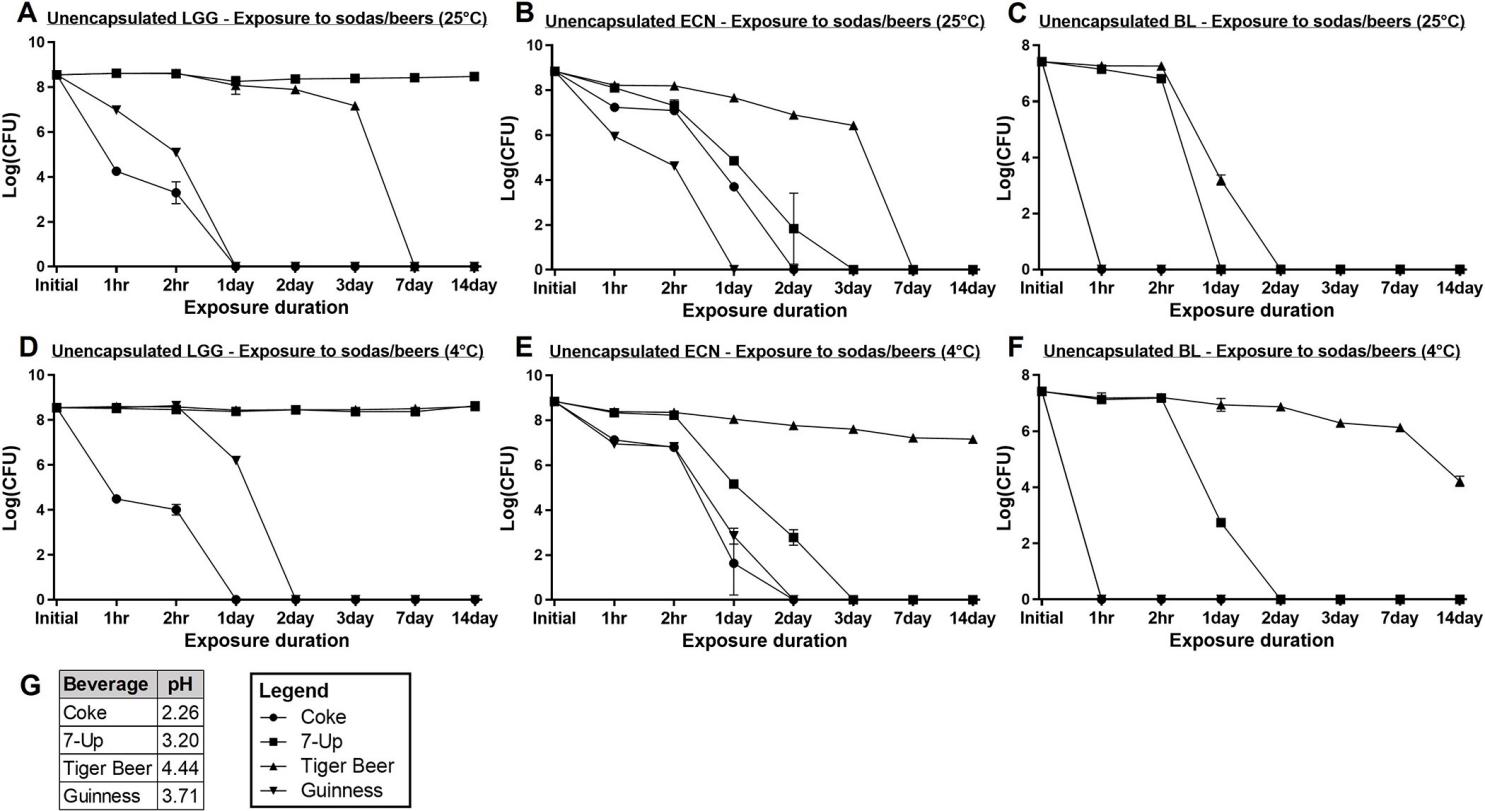
LogCFU survival of unencapsulated probiotics in various beverages at room temperature and refrigerated conditions for up to 14 days. (a) Unencapsulated LGG survival at 25°C, (b) unencapsulated ECN survival at 25°C, (c) unencapsulated BL survival at 25°C, (d) unencapsulated LGG survival at 4°C, (e) unencapsulated ECN survival at 4°C, and (f) unencapsulated BL survival at 4°C; (g) pH values of various beverages measured at room temperature.

Various probiotics were directly exposed to sodas and beers to determine their intrinsic survivabilities in these beverages. From **Fig 1**, varying extents of cell death were observed for the different probiotics at different exposure temperature conditions. Unencapsulated LGG showed the greatest susceptibility to Coke, with ~ 5 logCFU reduction following two hours of exposure at room temperature and refrigerated conditions (**Fig 1A** and **1D**). LGG was also highly susceptible to Guinness, with complete viability losses observed within < 1 day exposure at room temperature, and < 2 days in refrigerated conditions. For exposure to Tiger Beer, unencapsulated LGG was able to survive up to the 14 days tested in refrigerated conditions with no viability losses, but saw complete death within seven days at room temperature conditions. Unencapsulated LGG was able to survive and maintain its viability in 7-Up at both temperature conditions throughout the 14 days of exposure.

ECN similarly showed the greatest susceptibility to Coke and Guinness, with complete viability losses in both beverages at room temperature and refrigerated conditions within < 2 days of exposure (**Fig 1B** and **1E**). 7-Up exposure also demonstrated significant antagonism to unencapsulated ECN survival, resulting in complete cell death within < 3 days of exposure at both temperature conditions. Amongst the four tested beverages, ECN was most tolerant to the Tiger Beer media, however, 1.6 logCFU losses were observed after 14 days of refrigerated exposure, and at room temperature, ECN did not survive beyond seven days.

Unencapsulated BL experienced significant viability losses in all four tested beverages (**Fig 1C** and **1F**). Coke and Guinness were most antagonistic to BL, with complete cell death observed within 1 hour of exposure at both room temperature and refrigerated conditions. For 7-Up exposure, BL showed complete susceptibility within < 1 day exposure at room temperature, and < 2 days in refrigerated conditions. Tiger Beer resulted in complete death of BL within < 2 days exposure at room temperature, and 3.2 logCFU losses after 14 days of refrigerated exposure.

Overall, all three unencapsulated probiotics, LGG, ECN, and BL, showed better survival in 4°C refrigeration than at 25°C room temperature conditions. Lower storage temperatures are known to generally improve microorganism survival due to reduced rates of metabolism and chemical reactions, which can slow the effects of protein degradation or cell membrane oxidation [21, 22]. Comparing LGG, ECN, and BL, LGG generally showed better survival in the sodas and beers than ECN and BL. This may be attributed to the better acid and oxygen tolerance of LGG. LGG is a specifically isolated probiotic strain with an ability to proliferate at acidic conditions of pH 3 and higher [23], hence is expected to have higher resistance to the acidic beverages tested. Lactobacilli, like LGG, are also generally more tolerant to oxygen than *Bifidobacteria*, as the former are facultative anaerobes, while the latter are obligate anaerobes [3]. In addition, lactobacilli and other Gram-positive bacteria are known to exhibit acid resistance mechanisms to enhance their survival in acidic conditions, such as via proton exclusion mechanisms in the presence of metabolizable sugars [24, 25]. This may have provided LGG with greater intrinsic ability to survive in acidic beverage conditions.

Comparing the two tested sodas, Coke and 7-Up, Coke was significantly more antagonistic to the survival of all three probiotics than 7-Up. This is likely due to the more acidic pH of Coke, which was measured at 2.26, as compared to 3.20 for 7-Up (**Fig 1G**). Between the two tested beers, Tiger Beer and Guinness, Guinness was more detrimental towards the survival of all three probiotics. Guinness is an ale-stout, which is produced in warmer conditions of 16 to 24°C by fermentation of *Saccharomyces cerevisiae*. On the contrary, Tiger Beer is a lager, which is produced in colder conditions of around 10°C by fermentation of *Saccharomyces pastorianus* [26]. Several studies have evaluated the composition of stouts and lagers; stouts are known to present higher concentrations of hops iso-α-acids, such as xanthohumol, isoxanthohumol, 6-prenylnaringenin, and 8-prenylnaringenin, than lagers, which likely contributed to stronger bactericidal effects in stout [26–28]. Hops iso-α-acids are known to have potent inhibitory effects towards Gram-positive bacteria, while Gram-negative strains tend to be more resistant against these compounds [29]. From **Fig 1B** and **1E**, ECN demonstrated susceptibility particularly to Guinness Beer. This may be due to a combined effect of hops iso-α-acids antimicrobials and the acidified pH of the beverage [30]. Guinness also contained a higher alcohol content (5.5% alcohol by volume (ABV)) as compared to Tiger Beer (5% ABV), which likely presented a more challenging environment for the survival of probiotics.

### 3.2 Survival of unencapsulated probiotics in SGF

**Fig 2:**
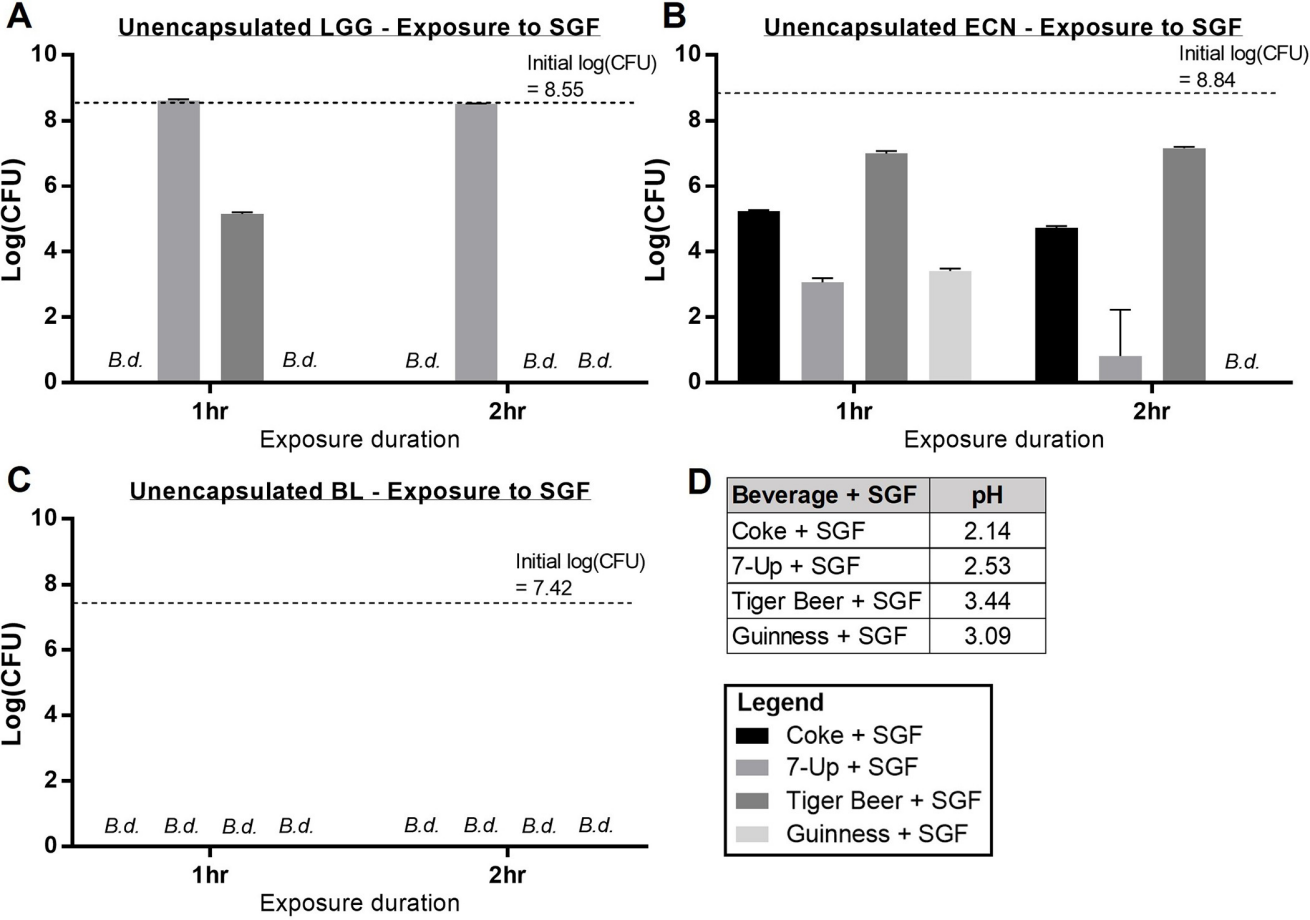
LogCFU survival of unencapsulated probiotics in various SGF and beverage combinations. (a) Unencapsulated LGG survival, (b) unencapsulated ECN survival, (c) unencapsulated BL survival; (d) pH values of various SGF and beverages combinations at room temperature. B.d. indicates cell count was below detection limit of ~1.5 logCFU.

The survivabilities of unencapsulated probiotics in SGF and beverage combinations are presented in **Fig 2**. All three probiotics, LGG, ECN, and BL, showed lower survival in beverage-SGF combinations, as compared to beverages alone. LGG showed complete viability losses following two-hour exposure in Coke-, Tiger Beer-, and Guinness-SGF solutions (**Fig 2A**), indicating that unencapsulated LGG would not survive gastric passage if it were to be consumed alongside the soda and beer beverages. ECN showed significant logCFU losses of 4.1, 8.0, 1.7, and 8.8 for Coke-, 7-Up-, Tiger Beer-, and Guinness-SGF solutions respectively, after two hour exposure (**Fig 2B**). Unencapsulated BL was completely susceptible towards all beverage-SGF combinations within one hour exposure (**Fig 2C**). Unsurprisingly, the poor survival of probiotics in beverage-SGF combinations is very likely attributed to the lower pH of the simulated gastric environment (**Fig 2D**). Upon comparison of Tiger Beer-SGF (pH 3.44) with 7-Up only (pH 3.20), it is also interesting to note that Tiger Beer-SGF was significantly more antagonistic than 7-Up towards the survival of all three probiotics, although the pH of Tiger Beer-SGF was higher. This suggests that a combinatory effect of bactericidal compounds present in Tiger Beer and the low pH due to the addition of SGF exacerbated survival of the probiotics in the Tiger Beer-SGF solution. Overall, the survival of all three unencapsulated probiotics in beverage-SGF combinations was unsatisfactory, and encapsulation formulations to improve their viability in these beverages and SGF are required.

### 3.3 Morphology of alginate-encapsulated beads

**Fig 3:**
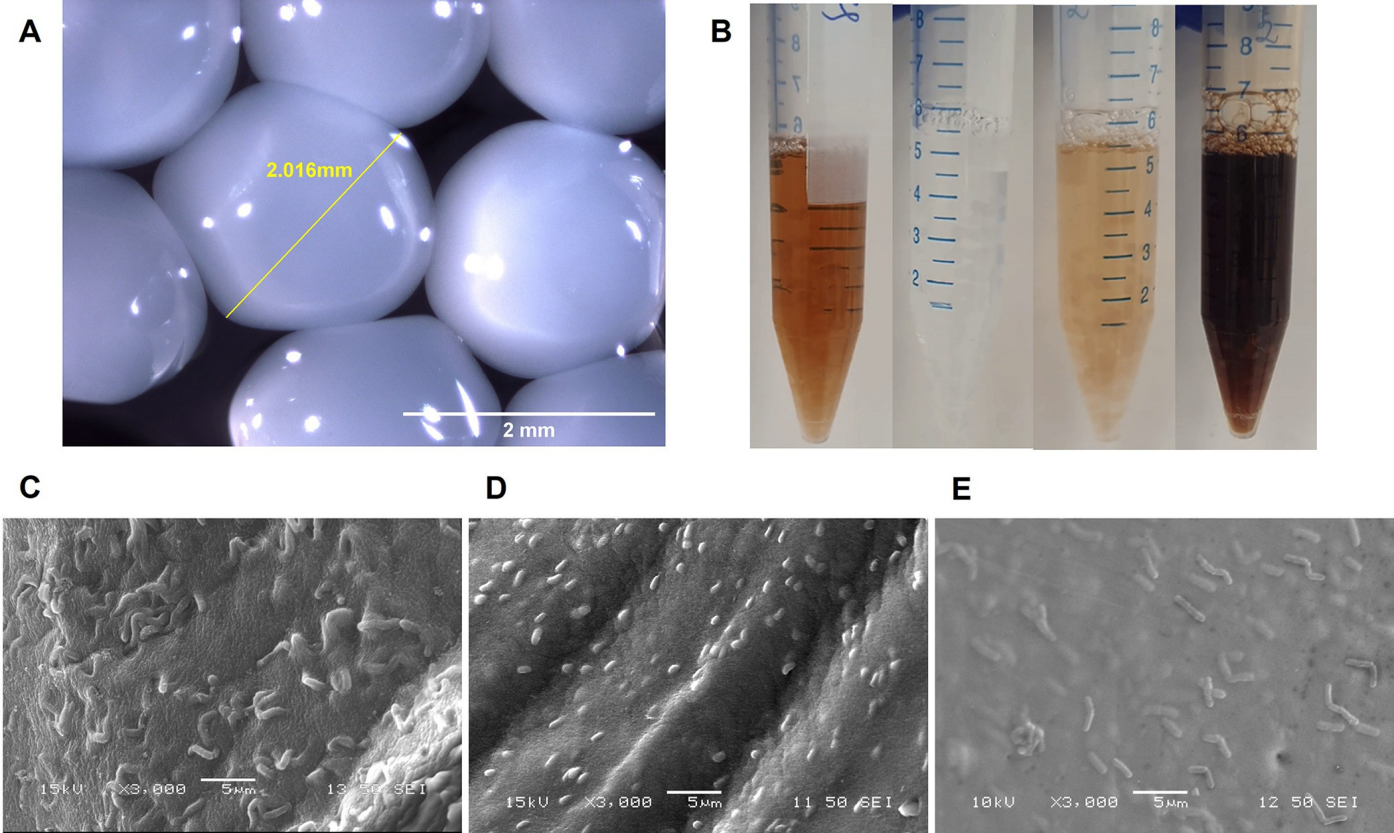
Images of alginate–encapsulated probiotic beads. (a) Light microscope images of alginate–encapsulated beads; (b) Alginate–probiotic beads added to various beverages, from left to right, Coke, 7–Up, Tiger Beer, and Guinness; SEM images of (c) LGG, (d) ECN, and (e) BL embedded within calcium–alginate beads.

Alginate encapsulation was performed, and **Fig 3** shows various images of the extruded probiotic-containing alginate beads. Extruded alginate beads appeared spherical and whitish, and were approximately 2 mm in diameter (**Fig 3A**). Upon incorporation into various beverages, the alginate-probiotic beads sank and appeared “boba-like” (**Fig 3B**). Various probiotics, LGG, ECN, and BL were observed to be embedded within the crosslinked calcium alginate matrix, suggesting that these probiotic bacteria were physically entrapped within the alginate matrix (**Fig 3C–3E**).

### 3.4 Encapsulated probiotics survival in beverages

**Fig 4:**
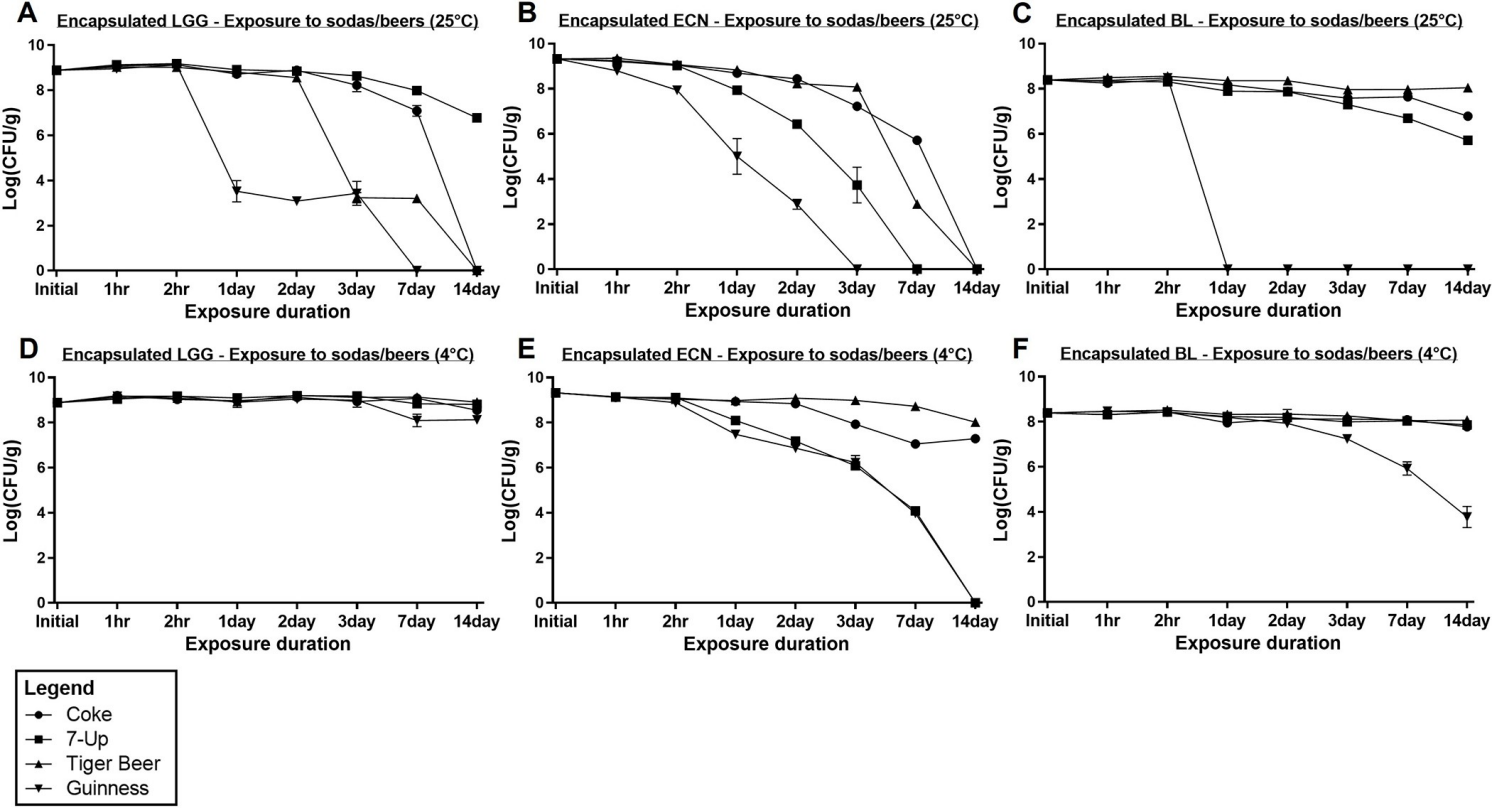
Encapsulated probiotics logCFU survival in various beverages at room temperature and refrigerated conditions for up to 14 days. (a) Encapsulated LGG survival at 25°C, (b) encapsulated ECN survival at 25°C, (c) encapsulated BL survival at 25°C, (d) encapsulated LGG survival at 4°C, (e) encapsulated ECN survival at 4°C, and (f) encapsulated BL survival at 4°C.

**Fig 5:**
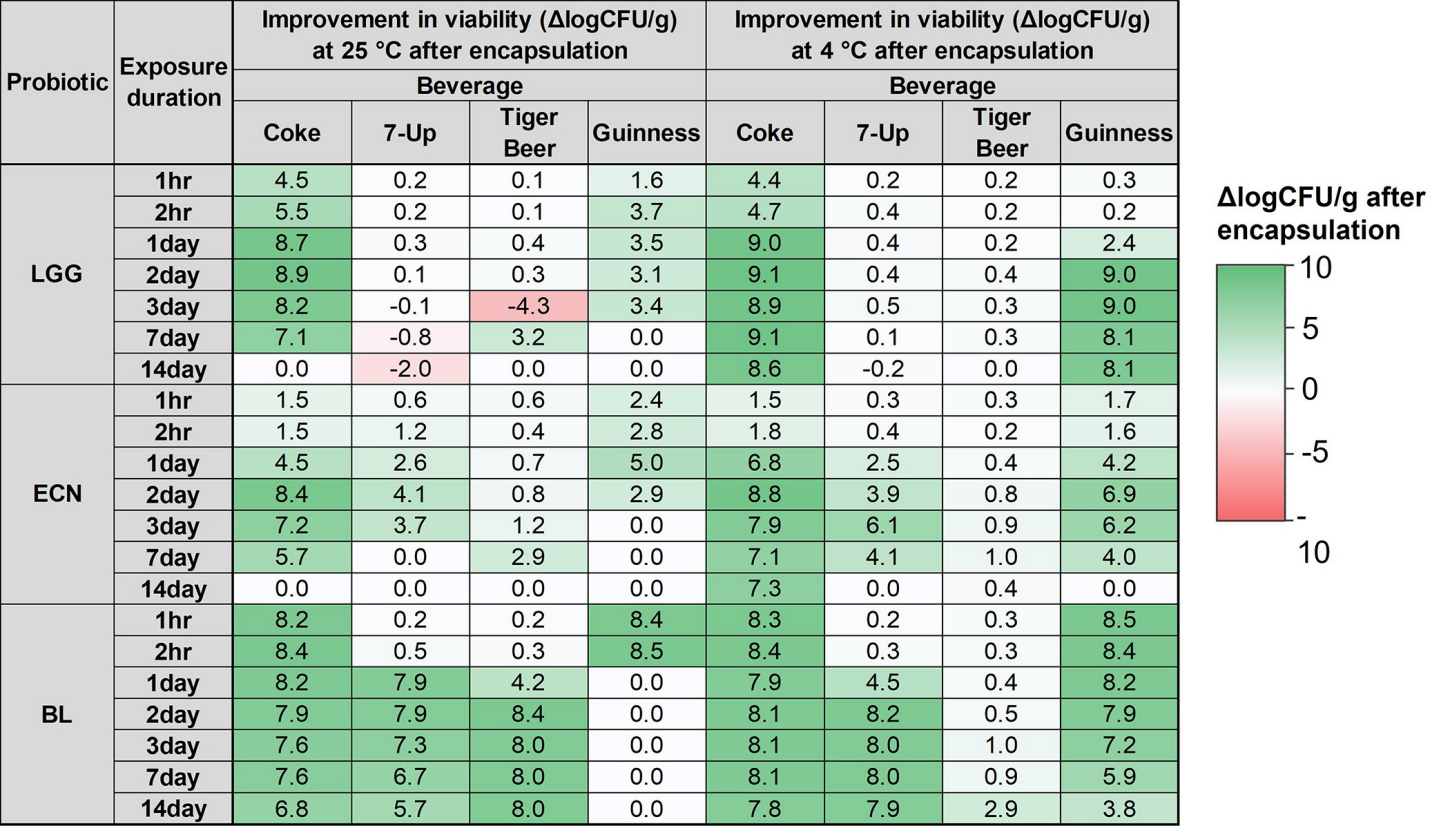
Heatmap showing improvement in probiotics viability after alginate encapsulation.

The encapsulated probiotics were exposed to various sodas and beers to determine their survivabilities. Comparing **Fig 1** with **Fig 4**, and with reference to **Fig 5**, there was a significant improvement in the survival of all three probiotics, LGG, ECN, and BL, upon encapsulation. Encapsulated LGG was observed to survive with < 1 logCFU/g losses for all beverages tested after 14 days of exposure in refrigerated conditions (**Fig 4D**). At room temperature conditions, the survival of encapsulated LGG was worse than at 4°C, but was considerably improved compared to unencapsulated LGG for Coke and Guinness exposure (**Fig 5**). Encapsulated LGG survived for up to two days of exposure to Coke at room temperature with < 1 logCFU/g loss, while unencapsulated LGG showed 5.3 logCFU losses within two hours of exposure (**Figs 1A** and **4A**). Additionally, encapsulated LGG maintained high viability with < 1 logCFU/g loss within two hours of Guinness exposure at room temperature, as compared to 3.5 logCFU losses for unencapsulated LGG.

For ECN, a significant improvement in survival was observed for encapsulated ECN exposed to various beverages within two hours of room temperature and refrigerated exposure, with > 8 logCFU/g remaining viable (**Figs 4B and 4E**, and **5**). At refrigerated conditions, encapsulated ECN also showed high survival (> 7 logCFU/g) in Coke and Tiger Beer after 14 days and > 6 logCFU/g survival in 7-Up and Tiger Beer after 3 days. On the other hand, at room temperature conditions, encapsulated ECN survived with > 7 logCFU/g in Coke and Tiger Beer after three days exposure, while encapsulated ECN saw significant viability losses (< 6 logCFU/g surviving) in 7-Up and Guinness following one day and three days exposure respectively.

Survival of BL was similarly enhanced upon alginate encapsulation (**Fig 4C, 4F**, and **Fig 5**). High survival of encapsulated BL was maintained (> 6 logCFU/g surviving) in Coke, 7-Up, and Tiger Beer after 14 days exposure at both temperature conditions. For Guinness exposure, high survival of encapsulated BL was achieved (> 8 logCFU/g) within two hours exposure at room temperature and one day exposure at refrigerated conditions.

For all three probiotic strains, the most pronounced improvement brought about by encapsulation was for Coke exposure (**Fig 5**), with > 7 logCFU/g improvement in viability for all three encapsulated probiotics after 14 days of refrigerated exposure. For 7-Up, a significant increase in survival was observed for encapsulated BL, with 7.8 logCFU/g remaining viable after 14 days of refrigerated exposure, as compared to complete viability losses of unencapsulated BL within two days of exposure (**Figs 1F** and **4F**). The improvement in viability could be attributed to the pH buffering capacity of alginate. In acidic pH, alginate is known to sequester protons and convert to alginic acid. Hence, alginate beads could maintain a localized pH higher than its surroundings, and this promoted the survival of encapsulated probiotics [14, 31], which were otherwise susceptible to the acidic pH in the carbonated sodas. The pH buffering mechanism likely improved survival of probiotics in Tiger Beer and Guinness as well. Various studies have demonstrated that the antibacterial effect of hops iso-α-acids increases with decreasing pH [30, 32]. With a higher localized pH, encapsulated probiotics were expectedly more tolerant to higher concentrations of hops iso-α-acids. Alginate encapsulation also likely reduced diffusivity of iso-α-acids and other inhibitory compounds present in beer, thereby prolonging the viability duration of encapsulated probiotics.

In general, it was observed that the viability of encapsulated probiotics was better maintained in 4°C refrigerated conditions as compared to at 25°C room temperature conditions throughout the 14 days test duration. Accordingly, in the development of probiotics encapsulation formulations for functional sodas and beers, the recommendation would be to store probiotics-incorporated beverages in refrigerated conditions to best preserve probiotics viability. Within the 14 days test duration, the best performing encapsulated probiotic and beverage combinations which yielded > 8 logCFU/g viable probiotics after refrigerated storage include LGG and all four tested beverages, ECN and Tiger Beer, BL and Coke/7-Up/Tiger Beer. Another alternative to incorporating probiotics within these sodas and beverages would be to develop alginate-probiotic beads which are stored separately from the beverages, only to be added into the beverages when ready to be consumed. In this case, the duration of exposure of encapsulated probiotics to the various beverages could be kept within two hours, where high viabilities of encapsulated probiotics (> 8 logCFU/g) could still be maintained.

### 3.5 Encapsulated probiotics survival in SGF

**Fig 6:**
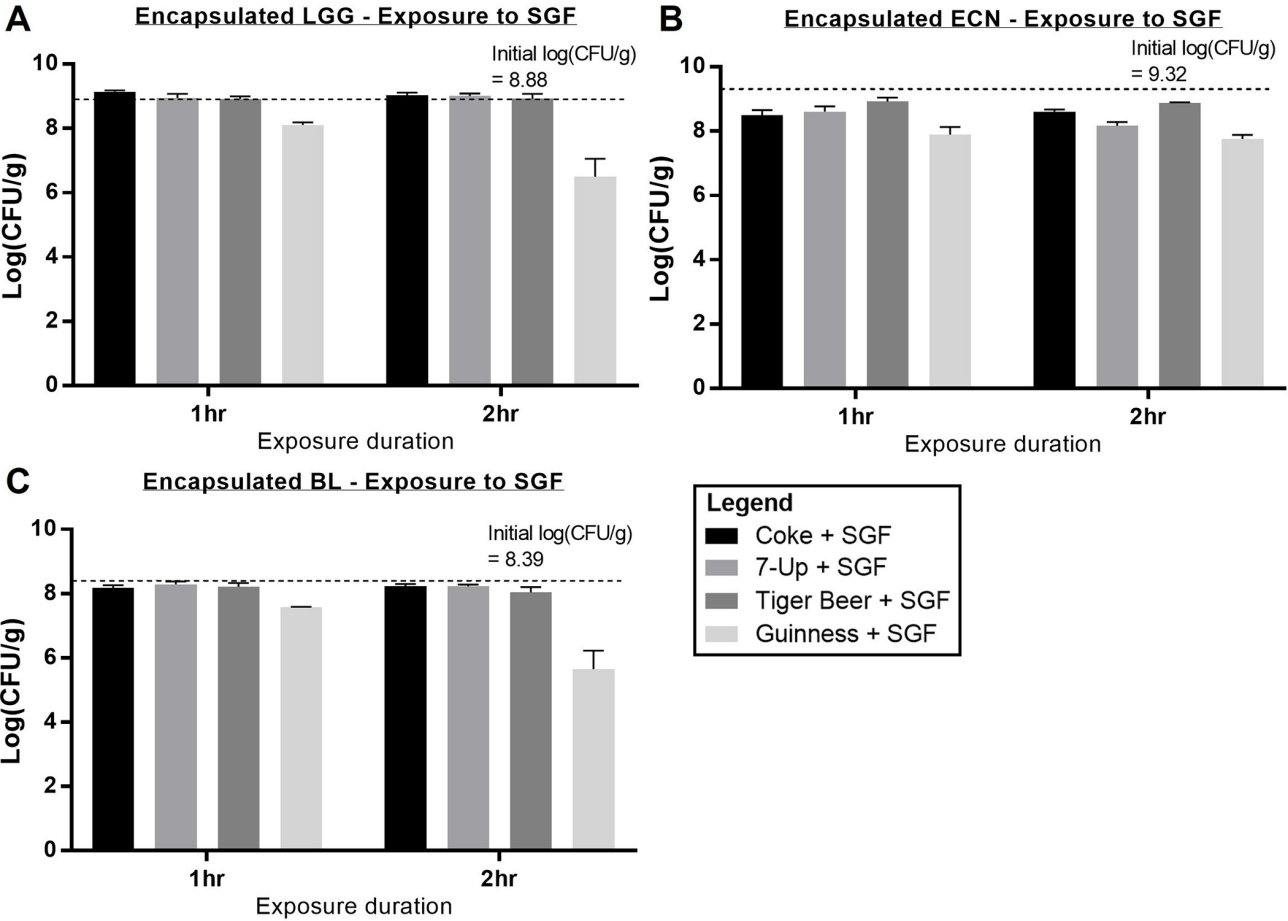
LogCFU/g survival of encapsulated probiotics in various SGF and beverage combinations. (a) Encapsulated LGG survival, (b) encapsulated ECN survival, and (c) encapsulated BL survival.

Survivabilities of all three probiotics were also significantly improved in SGF exposure conditions following alginate encapsulation (**Fig 2** and **Fig 6**). As seen in **Fig 6**, high viabilities (> 8 logCFU/g) were achieved following two hours of beverage-SGF exposure for LGG, ECN, and BL in Coke-, 7-Up-, and Tiger-SGF solutions, indicating that alginate encapsulation was able to protect these strains during gastric passage. For Guinness-SGF, 2.4, 1.6, and 2.7 logCFU/g reduction were observed for LGG, ECN, and BL respectively after 2 hours, however this was still a significant improvement as compared to unencapsulated probiotics. Overall, alginate encapsulation has demonstrated its utility in protecting probiotics within beverages, as well as during passage through the gastric environment.

### 3.6 Lactate production by LGG

The lactate content within LGG-containing beverages was studied to determine if lactate had accumulated as a by-product of LGG metabolism. An accumulation of lactate can potentially introduce undesirable “off-flavors” to beverages [19, 20]. Here, unencapsulated LGG and alginate-encapsulated LGG were exposed to various beverages and the lactate content was evaluated by HPLC. Increased L-lactate concentrations were detected in LGG-supplemented Coke and 7-Up, but not in Tiger Beer and Guinness. This is likely due to the absence of glucose in Tiger Beer and Guinness, as simple sugars tend to be depleted in beers during the yeast fermentation process [28].

**Fig 7:**
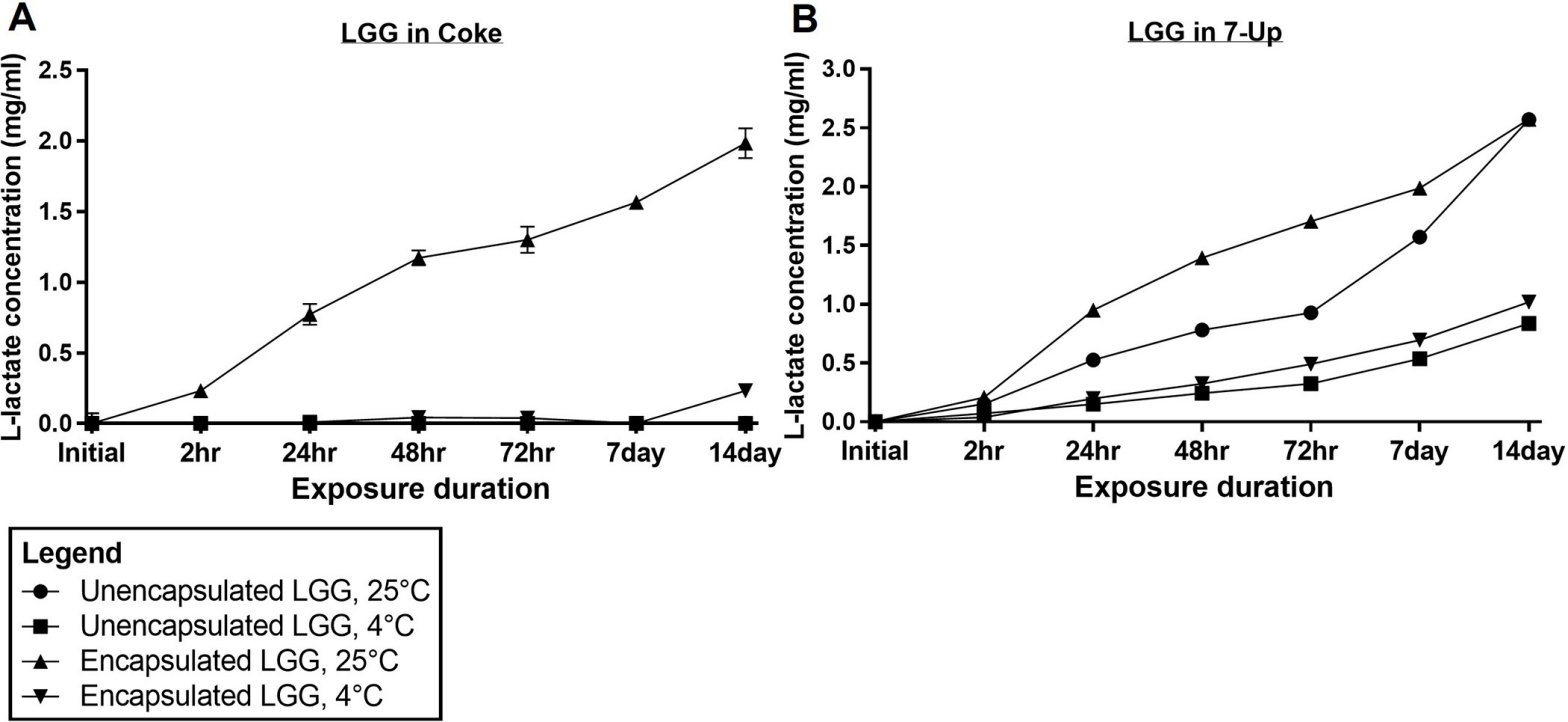
L–lactate concentration in various sodas with LGG added at room temperature and refrigerated conditions for up to 14 days. (a) LGG in Coke; (b) LGG in 7–Up.

From **Fig 7B**, an increase in L-lactate was observed in encapsulated LGG stored at room temperature conditions, with 1.98 mg/ml present following 14 days of exposure. A minor increase in L-lactate of 0.23 mg/ml was detected for encapsulated LGG in Coke stored at refrigerated conditions. No significant concentration of L-lactate was detected for unencapsulated LGG in Coke at both temperature conditions. For 7-Up, an increase in L-lactate was measured for both unencapsulated and encapsulated LGG at room temperature and refrigerated conditions (**Fig 7B**). L-lactate concentration reached 2.57 mg/ml for both unencapsulated and encapsulated LGG at room temperature conditions after 14 days, while encapsulated LGG in refrigerated conditions showed slightly higher L-lactate production of 1.02 mg/ml as compared to 0.84 mg/ml L-lactate produced by unencapsulated LGG. The data suggests that viable LGG was able to metabolize glucose present in Coke and 7-Up, and had produced a greater amount of L-lactate in 7-Up. With reference to the nutritional labels of these two sodas (**S1 Table in S2 File**), Coke used in this experiment had a higher concentration of total sugars (10.6 g/100ml) than 7-Up (4.7 g/100ml). Hence, the higher L-lactate concentration in 7-Up was not due to the concentration of sugars, but was likely due to the higher pH level in 7-Up, which provided a less hostile environment for LGG metabolism. Presently, there appears to be no released guidelines regarding the concentration of lactate which induces an “off-flavor”, as this depends on the type of beverage, and the relative amounts of other flavor compounds present in the beverage. Overall, further sensory studies are required to determine if the addition of lactobacilli, such as LGG, to soda beverages would result in significant organoleptic changes. Generally, the recommendation would be to store beverages, particularly those containing simple sugars which are fermentable by viable probiotics, under refrigerated conditions, to minimize unanticipated changes to beverage tastes.

## 4. Conclusion

This article reports the viability of unencapsulated and alginate-encapsulated probiotics exposed to various commercialized carbonated sodas and beers, including Coke, 7-Up, Tiger Beer, and Guinness. Three probiotic strains, LGG, ECN, and BL, were encapsulated in calcium-alginate spheres via extrusion and exposed to sodas and beers under refrigerated, room temperature and SGF conditions. Results showed that the viabilities of all three probiotics, both unencapsulated and encapsulated, were better maintained in refrigerated conditions as compared to room temperature, and LGG generally showed better survival in various beverages than ECN and BL Upon alginate encapsulation, survival of all three probiotic strains were improved in all tested beverages, most significantly in Coke, under all exposure conditions. Within the 14 days test duration, encapsulated probiotic and beverage combinations which yielded > 8 logCFU/g viable probiotics after refrigerated storage include, LGG and all four tested beverages, ECN and Tiger Beer, BL and Coke/7-Up/Tiger Beer. The effectiveness of alginate encapsulation in enhancing probiotic viabilities in these beverages can be attributed to the pH buffering effect of alginate in acidic media, which protects encapsulated probiotics from the acidic soda beverage, and mitigates antagonistic effects of acid in combination with bactericidal compounds present in beers (e.g., hops iso-α acids). Results also demonstrated that LGG added to carbonated sodas, Coke and 7-Up, can result in an accumulation of lactate as a by-product of sugar metabolism, and more lactate was produced at room temperature storage than refrigerated storage. Overall, it is recommended to store encapsulated probiotics under refrigerated conditions to reduce viability losses, and to avoid flavor alterations that could arise due to probiotic metabolism.

## Supporting information

S1 File (DOCX)

S2 File (DOCX)
