# Supplementary material for: Alginate encapsulation improves probiotics survival in carbonated sodas and beers
Source: PLoS One. 2023 Mar 31;18(3):e0283745. doi: 10.1371/journal.pone.0283745 (PMC10065269; doi:10.1371/journal.pone.0283745)
Supplement: S1 File — (DOCX) [file pone.0283745.s001.docx]

**Full Title**

Alginate Encapsulation Improves Probiotics Survival in Carbonated Sodas and Beers

**Short Title**

Encapsulated Probiotics in Carbonated Sodas and Beers

**Li Ling Tan^1^, Kai Lin Ang^1^, Say Chye Joachim Loo^1,2,3*^**

^1^School of Materials Science and Engineering, Nanyang Technological University, 50 Nanyang Avenue, 639798 Singapore

^2^Singapore Centre for Environmental Life Sciences Engineering (SCELSE), Nanyang Technological University, 60 Nanyang Drive, 637551 Singapore

^3^Lee Kong Chian School of Medicine, Nanyang Technological University, 11 Mandalay Road, 308232 Singapore

***Correspondence:**

Say Chye Joachim Loo

Email: [joachimloo@ntu.edu.sg](mailto:joachimloo@ntu.edu.sg)

**Supplementary Information**

Ingredient profile of tested beverages


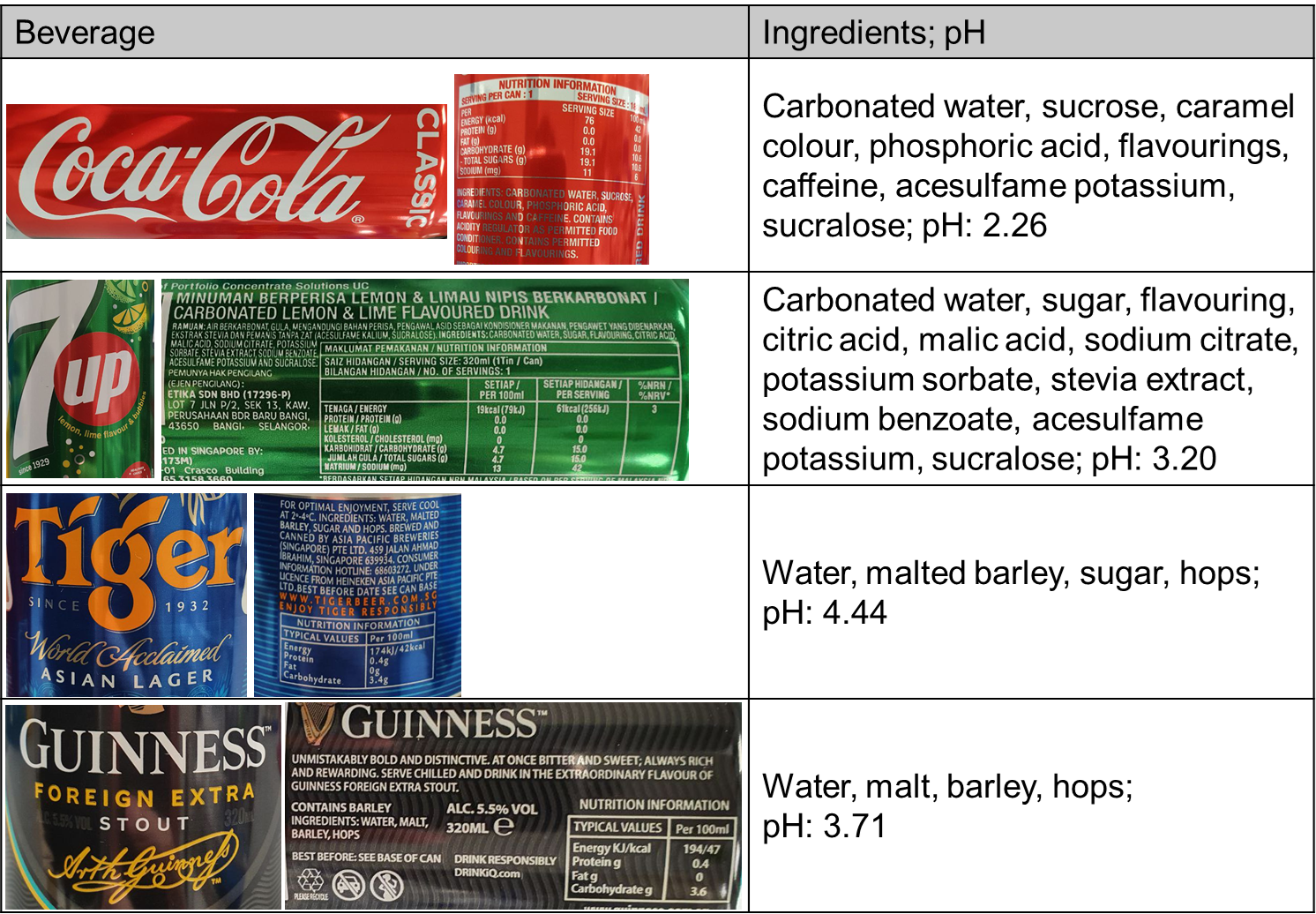


**Fig. S1.** Nutrition information and ingredient list of four tested beverages, Coke, 7-Up, Tiger Beer, and Guinness.​

Quantification of L-lactate by HPLC-RID


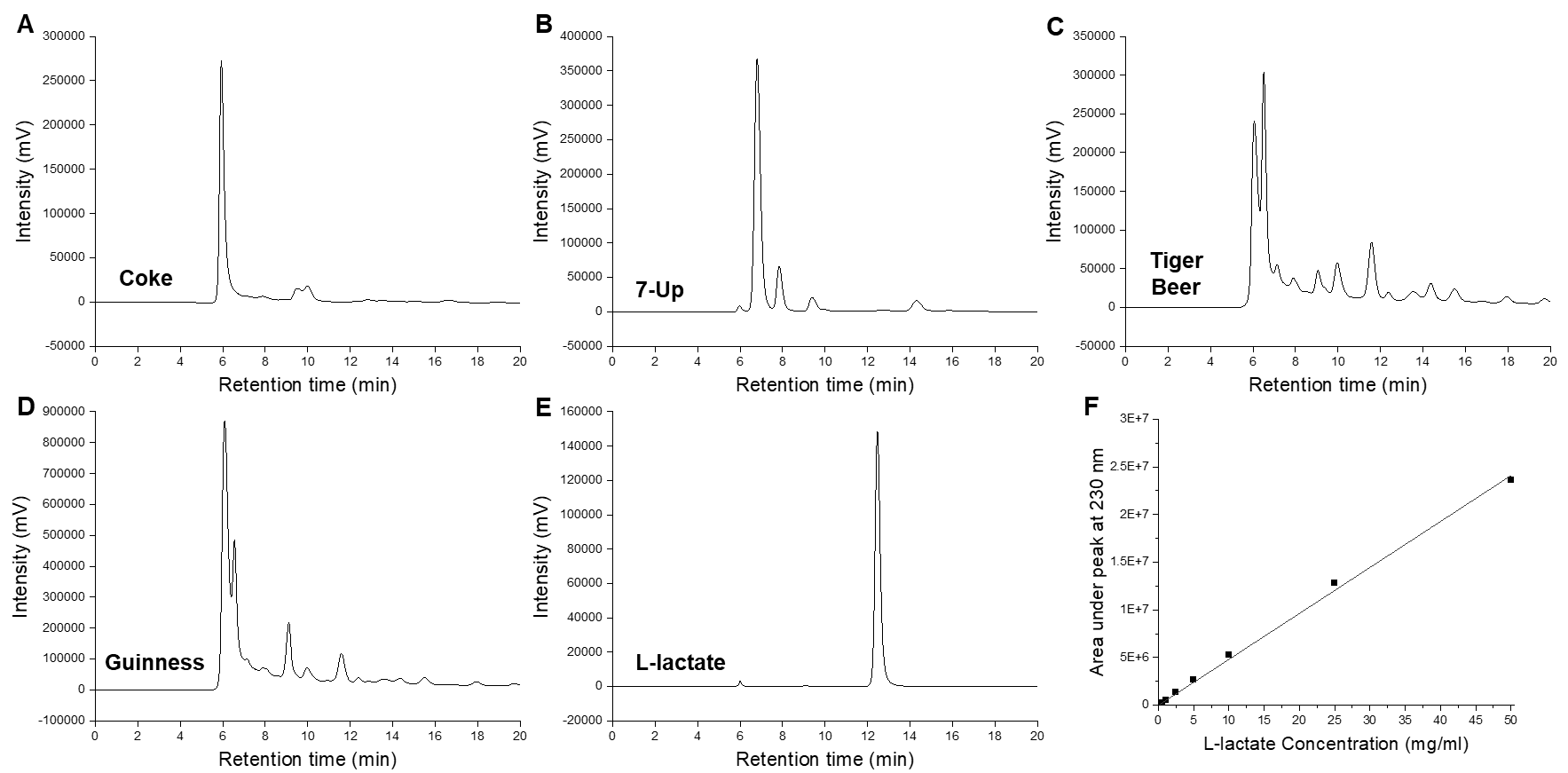


**Fig. S2.** HPLC chromatograms of (a) Coke, (b) 7-Up, (c) Tiger Beer, and (d) Guinness. The L-lactate chromatogram is presented as (e), while the standard curve as (f).
