# Supplementary material for: Alginate encapsulation improves probiotics survival in carbonated sodas and beers
Source: PLoS One. 2023 Mar 31;18(3):e0283745. doi: 10.1371/journal.pone.0283745 (PMC10065269; doi:10.1371/journal.pone.0283745)
Supplement: S2 File — (DOCX) [file pone.0283745.s002.docx]

Alginate encapsulation improves probiotics survival in carbonated sodas and beers

**Li Ling Tan^1^, Kai Lin Ang^1^, Say Chye Joachim Loo^1,2,3*^**

^1^School of Materials Science and Engineering, Nanyang Technological University, Singapore

^2^Singapore Centre for Environmental Life Sciences Engineering (SCELSE), Nanyang Technological University, Singapore

^3^Lee Kong Chian School of Medicine, Nanyang Technological University, Singapore

***Correspondence:**

Email: [joachimloo@ntu.edu.sg](mailto:joachimloo@ntu.edu.sg) (SCJL)

**Supplementary Information**

Ingredient profile of tested beverages

**Table S1.** **Nutrition information and ingredient list of four tested beverages, Coke, 7-Up, Tiger Beer, and Guinness.**

| Beverage | Ingredients; pH |  |
| --- | --- | --- |
| Coca-Cola | Carbonated water, sucrose, caramel colour, phosphoric acid, flavourings, caffeine, acesulfame potassium, sucralose; pH: 2.26 |  |
|  |  |  |
|  |  |  |
| 7-Up | Carbonated water, sugar, flavouring, citric acid, malic acid, sodium citrate, potassium sorbate, stevia extract, sodium benzoate, acesulfame potassium, sucralose; pH: 3.20 |  |
|  |  |  |
|  |  |  |
| Tiger | Water, malted barley, sugar, hops; pH: 4.44 |  |
|  |  |  |
|  |  |  |
| Guinness | Water, malt, barley, hops; pH: 3.71 |  |
|  |  |  |
|  |  |  |

Quantification of L-lactate by HPLC-RID


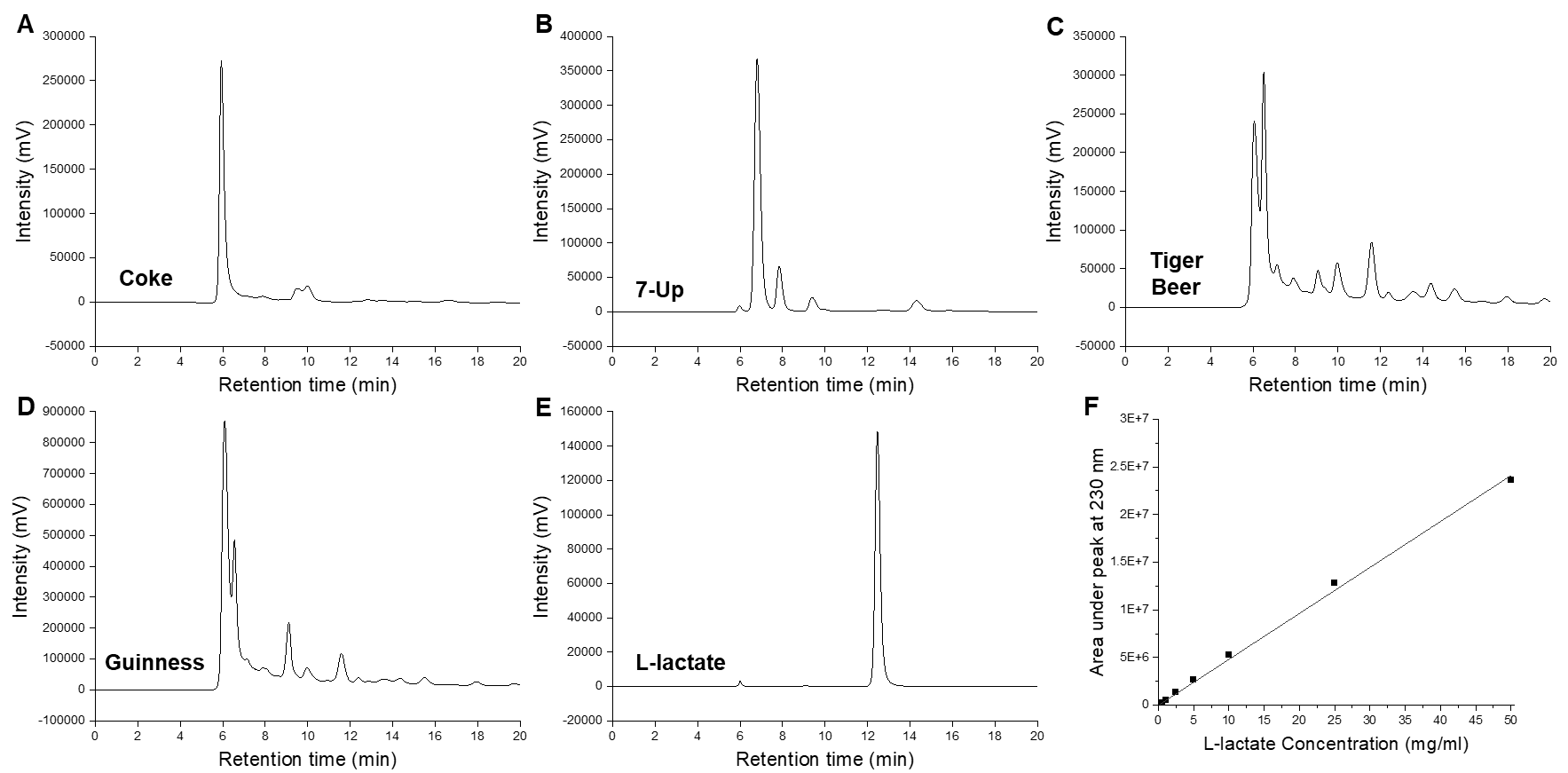


**Fig S1.** **HPLC chromatograms of (a) Coke, (b) 7-Up, (c) Tiger Beer, and (d) Guinness.** The L-lactate chromatogram is presented as (e), while the standard curve as (f).
